# The quest for multifunctional and dedicated PET instrumentation with irregular geometries

**DOI:** 10.1007/s12149-023-01881-6

**Published:** 2023-11-12

**Authors:** Amirhossein Sanaat, Mehdi Amini, Hossein Arabi, Habib Zaidi

**Affiliations:** 1 Division of Nuclear Medicine and Molecular Imaging, Geneva University Hospital, CH-1211 Geneva, Switzerland; 2 Department of Nuclear Medicine and Molecular Imaging, University of Groningen, University Medical Center Groningen, 9700 RB, Groningen, The Netherlands; 3 Department of Nuclear Medicine, University of Southern Denmark, 500, Odense, Denmark; 4 University Research and Innovation Center, Óbuda University, Budapest, Hungary

**Keywords:** Dedicated PET, Brain PET, Prostate PET, Breast PET, Cardiac PET

## Abstract

We focus on reviewing state-of-the-art developments of dedicated PET scanners with irregular geometries and the potential of different aspects of multifunctional PET imaging. First, we discuss advances in non-conventional PET detector geometries. Then, we present innovative designs of organ-specific dedicated PET scanners for breast, brain, prostate, and cardiac imaging. We will also review challenges and possible artifacts by image reconstruction algorithms for PET scanners with irregular geometries, such as non-cylindrical and partial angular coverage geometries and how they can be addressed. Then, we attempt to address some open issues about cost/benefits analysis of dedicated PET scanners, how far are the theoretical conceptual designs from the market/clinic, and strategies to reduce fabrication cost without compromising performance.

## Introduction

During the past 70 years or so, since the invention of the first positron-emitting imaging device in Massachusetts General Hospital, Boston [1], PET instrumentation evolved drastically in terms of performance characteristics, application, and availability. It is evident that improving the performance of PET in terms of spatial resolution and sensitivity will lead to wider adoption of this imaging modality in the clinic. Yet, an increase in the number of reimbursed clinical indications does not necessarily lead to higher global availability and accessibility to this technology. Availability/Accessibility depends mostly on fabrication cost. Based on a recent report by the International Atomic Energy Agency (IAEA), among 212 countries, only 109 have access to PET technology [2]. The number of PET scanners in high-income countries is 3.52 per million population, while it falls to 0.004 per million in low-income countries. Gallach et al. showed that at least 96 countries need to increase the number of PET/CT scanners and more than 200 additional PET/CT scanners are necessary to address the main common types of cancer, including lung, colorectal, lymphoma, head and neck, melanoma, and esophagus [3]. They estimated that approximately 229.3 M US$ are needed to equip these 96 countries with 16-slice PET/CT scanners. These statistics raise a few important questions; should the medical physics community (mainly instrumentation research groups) focus on improving PET scanners’ performance or their accessibility? Is it really necessary to compete toward developing fancy detector modules or complex PET configurations to improve the spatial resolution by a few percent or compete on developing methods for reducing fabrication cost? As a thought experiment, is it more beneficial for the society to have more PET scanners with low performance or fewer PET scanners with high performance?

A dedicated or organ-specific PET scanner may be the answer to the above-mentioned questions. Dedicated PET scanners optimized for scanning one specific organ offer both high performance and low manufacturing cost in comparison with general-purpose high-end whole-body PET scanners, which makes them more affordable and accessible. Their easier commissioning, maintenance, and training in addition to their smaller fingerprint or space consumption make them ideal for low-income and middle-income countries and small clinics in high-income countries. Although dedicated PET scanners inherently bear limitations in common clinical scenarios requiring whole-body scans (e.g., staging in clinical oncology), it must be emphasized that these specialized scanners, though more accessible, are not intended to replace the broader utility of whole-body PET scanners in clinical setting.

Apart from organ-specific dedicated PET scanners, designing irregular whole-body PET scanners is of great significance in the field of PET instrumentation. Improving the design of whole-body PET scanners by introducing novel detector concepts and geometrical configurations holds a significant level of enthusiasm in applied research. Novel ideas include extendable axial field-of-view [4], adjustable gantry diameter/shape [5], or scanners equipped with thick and thin detectors’ modules [6], or using plastic scintillators [7] and pseudo-pixelated crystals [6]. Besides the novelties in PET hardware, software advances, specifically involving the use of Artificial Intelligence (AI), might play a crucial role in reducing fabrication costs and improving PET performance. A number of preliminary studies have shown that AI have the potential to reduce cost and complexity, for instance, by removing the time-of-flight (TOF) hardware [8, 9], CT for attenuation correction [10], or even improving the resolution of monolithic and pixelated crystals [11]. In this review, we describe irregular and dedicated PET scanners and discuss the technical innovations that are likely to drive the future of conventional PET scanners. This introductory paper summarizes briefly recent advances in the field and provide insights on potential future developments.

## Advances in non-conventional PET detector geometries

Research on PET detectors, at both the hardware and software levels, has mostly been focused on improving the key performance characteristics of the detectors, namely, the spatial resolution and the intrinsic sensitivity. Increasing the detector's sensitivity elevates the collected true coincidence events at a decreased level of injected activity and shortened scanning time, whereas achieving better spatial resolution improves image quality and quantitative accuracy. On one hand, using thicker crystals allows higher sensitivity at the cost of reduced depth-of-interaction (DOI) localization accuracy, which leads to parallax errors [inaccuracy in positioning the line of response (LOR)] and deteriorated spatial resolution. On the other hand, higher spatial resolution is achievable with smaller crystal cross-sectional size, but this will worsen detector sensitivity due to poor scintillation light collection and crystal identification. Accordingly, there is an intrinsic trade-off between key characteristics of the PET detectors making technological advancements challenging [12].

The solution to this trade-off is substantiated in two key technologies, namely, DOI determination and TOF capability [13]. The DOI information minimizes the parallax error and allows providing a more uniform spatial resolution. This is gaining importance in organ-specific imagers with small gantries and/or irregular geometries [14, 15]. Last but not least, DOI might help improving the energy resolution by generating specific photopeaks for different depths of annihilation photons’ detection when the crystals have a rough surface finish [16]. Furthermore, TOF information has the potential to significantly increase the signal-to-noise ratio (SNR) of PET images by limiting the location of the positron annihilation point along the LOR to a smaller segment [17]. High temporal resolution TOF technology is being increasingly highlighted in recent PET instrumentation research. Although currently, coincidence time resolutions (CTR) below 150 picoseconds (ps) full width-at-half-maximum (FWHM) are challenging to obtain, the ultimate goal is 10 ps CTR FWHM, since theoretically, it would directly give access to a reasonably accurate position of the positron annihilation [18]. This information enables to alleviate the difficulties associated with image reconstruction. This is particularly important in organ-specific PET scanners owing to the multiple challenges introduced by irregular geometries in the reconstruction process. A couple of scanner manufacturers unveiled at the last annual meeting of the Society of Nuclear Medicine (June 2023) novel PET scanners achieving temporal TOF resolutions of 178 ps and 194 ps for Siemens Healthineers and United Imaging, respectively.

However, TOF and DOI measurements are not independent of each other. The uncertainty in the DOI can induce errors in timing resolution due to the speed of optical photons in dense crystal medium. In Table 1, we summarized the most innovative detector designs by considering important aspects, such as energy, DOI, TOF resolution, and type of scintillator and readout technology. We also summarized the golden innovation aspects of these studies in the innovation column. Common techniques include Phoswich detectors [19, 20] equipped with pulse-shape discrimination schemes [21], multiple-layered detectors with independent readout for each layer [22], dual-ended readout techniques [23, 24], light-sharing detectors using a particular arrangement of crystals and reflectors [13, 25, 26], detectors with phosphor-coated crystals [27], sub-surface laser-induced optical barriers [28, 29], monolithic crystals coupled with a retroreflector layer [30], and finally machine learning approaches [11, 31, 32].

**Table 1:** Summary of the most important/novel developments of detector modules

Name	Year	DOI	TOF (ps)	Energy resolution	Scintillator	Crystal type	Crystal dimension (mm^3^)	Readout	Innovation
Phoswich PET detector [20]	1999	Accuracy: 86% for LSO 80% for GSO 84% for BGO	_	19% for LSO 21% for GSO 40% for BGO	LSO GSO BGO	pixelated	2 × 2 × 4	single-ended PSPMT	Three-layer Phoswich PET detector modules
Dual layer modular detector [127]	2003	–	–	–	BGO	pixelated	1.98 × 1.98 Thickness = 6.5 and 11.5	PSPMT	They pixelate the crystal from cutting a relatively large block into a dual-layer pseudo discrete pattern.
Four-layer DOI detector [128]	2004	–	–	11.2–13.7%	Gd_2_SiO_5_	pixelated	1.42 × 1.42 × 4.5	PSPMT	special reflector arrangement was used for each layer to improve DOI
Dual-sided readout DOI-PET module [129]	2013	A: 3 mm B: 5 mm	_	A: 9.8 ± 0.8%, B: 11.8 ± 1.3%	Ce:GAGG	pixelated	A: 3 × 3 × 3 B: 0.8 × 0.8 × 5	double-sided MPPC	DOI-PET detector based on monolithic crystals and dual-ended readout
Stacked-crystal PET detector [19]	2015	Accuracy: 91–98%	153 ps 199 ps	_	LaBr^3^ CeBr^3^	pixelated	1 × 1 × 2 thickness = 12 and 15 mm	PMTSiPM	2-layer detectors based on several configurations of LaBr3 scintillators, with different Cerium dopant concentrations, read by PMTs and SiPMs The stacked scintillator structure reveals a specific signal for events in each layer, which is utilized to assign the DOI
DOI-PET module with dual-ended readout and SSLE crystals [130]	2018	A: 3 mm B: 1.5 mm	A:783 ps B: 1.14 ns	A: 10.1% B: 10.8%	LFS	pixelated	A: 3 × 3 × 20 (7 depth segments), B: 1.5 × 1.5 × 20 (13 depth segments)	dual-ended MPPC	DOI detectors with crystal bars segmented using sub surfaced laser engraved (SSLE) techniques and dual-ended readout scheme. Anger calculation was used to obtain a three-dimensional map of position of the detectors
Light-sharing DOI-TOF-PET detector [25]	2019	3 mm	157 ps	9%	LYSO:Ce	pixelated	1.53 × 1.53 × 15	Single-ended SiPM	extracting DOI info with light-sharing technique between the crystals with recirculation mechanism, with a specific focus on timing performance of the detectors
Polaroid-PET*, [131]	2020	0.49–1.06 mm	_	_	LYSO	Monolithic	50.2 × 50.2 × 10	Single-ended SiPM	polaroid inserted between the crystal and SiPM to reduce reflection effect
Prismatoid light guide PET [26]	2020	2.5 mm	254	9%	LYSO	pixelated	1.4 × 1.4 × 20	Single-ended SiPM	Single-ended readout TOF-DOI-PET detector with light-sharing technique using prisms
DOI-PET detector based on quadrisected crystals [132]	2020	_	_	9.10%	GAGG	pixelated	1.45 × 1.45 × 4.5	Single-ended MPPC	light-sharing single-end readout DOI with quadrisected crystals in 4-layers using Anger calculation, responses of all the crystal elements were distinguished on a 2D positioning histogram map
TOF-PET detector [133]	2021	_	107 ± 3 ps	10.5%	LSO	Pixelated	1.9 × 1.9 × 10	Side readout SiPM	TOF-PET detector by coupling two crystals with different decay times, and reading them in a side readout scheme
Crosshair light-sharing module with GFAG crystal [13]	2021	4.7 mm	402 ps	14%	GFAG	pixelated	1.45 × 1.45 × 20	Single-ended MPPC	light-sharing single-end readout DOI-PET detector with continues layered crystals
Crosshair light-sharing module with LGSO crystal [33]	2021	4.7 mm	293 ps	10%	LGSO	pixelated	1.45 × 1.45 × 15	Single-ended MPPC	light-sharing single-end readout DOI-PET detector with continues layered crystals
DOI-TOF semi-monolithic PET detector	2022	2.12 mm	209 ps	11.30%	LYSO	Semi Monolithic	3.9 × 32 × 19	Single-ended SiPM	DOI-TOF semi-monolithic crystals with the goal of combining the advantageous of both pixelated and monolithic detectors
DOI-TOF-PET detector [31]	2022	1.2 mm	156 ps	_	LYSO	Monolithic	25 × 25 × 8	SiPMs	monolithic PET detectors with DOI and TOF information extracted using AI

*Represents simulation studies

The Phoswich detectors’ approach commonly consists of multiple layers of different types of scintillators with different decay times, stacked on each other. Although Phoswich detectors can achieve good DOI resolution, their timing resolution is degraded. In fact, the boundaries between the layers reduce the number of optical photons arriving at the photosensors, and the variability of the arrival time of photons from different layers degrades the timing resolution [13]. Light-sharing detectors couple two crystals together by a particular arrangement of reflectors to imitate a dual-ended readout with a single-ended design. Pizzichemi et al. developed a TOF-DOI-PET module containing an array of crystals with 4-to-1 crystal-to-SiPM arrangement at one end, and a uniform glass light guide on the opposite side to redirect upgoing photons back into neighboring crystals [25]. By using particular prisms for crystals at edges and corners, and optimizing inter-crystal light sharing due to the prism reflection, LaBella et al. achieved better crystal identification, DOI resolution of 2.5 mm, and energy resolution of 9% [26].

Another series of light-sharing detectors, known as crosshair light-sharing detectors [13, 33], consist of crystal pairs partially coupled with optical windows, attached to two different Multi-Pixel Photon Counters (MPPCs). Parts of the crystals that are not coupled with optical windows are attached to reflectors. The DOI and crystal identification is calculated based on the output pattern of the paired MPPCs.

Detector modules based on monolithic crystals have a number of advantages, such as higher sensitivity, the ability to extract DOI, no zero detection regions, decent performance in spatial resolution, and less manufacturing cost. However, these detectors commonly require complex calibration procedures, and complicated algorithms for the location, energy, and timing assignation of photon interactions. Moreover, the spatial resolution deteriorates around the edges, although multiple studies attempted to confront this issue by calibrating the detector using analytical [34], simulation-based [35], and experimental [36] approaches.

Various research groups developed semi-monolithic detectors based on different designs. Sabet et al. [29] proposed a semi-monolithic detector using laser-induced optical barriers (LIOB), which creates small defects inside the LYSO crystal bulk that operate as an optical reflector, to combine the advantages of monolithic and pixelated crystals. Sanaat et al. suggested a novel concept for deflecting the trajectory of optical photons passing through a monolithic scintillator [37]. The proposed technique consists of a reflective belt created from millions of optical barrier points covering the surroundings of the crystal, created by the LIOB method. A monolithic crystal with a belt of reflectors created by laser engraving can lead to better spatial resolution and sensitivity.

Most recently, artificial intelligence was introduced as an effective tool for both accurate TOF estimation and positioning of photon interactions in PET detectors [38–40]. The best performance for both event positioning and time stamping resolution have been attained by complex algorithms, such as gradient tree boosting [41], maximum-likelihood [41], nearest neighbors [42], and neural networks [43] applied on monolithic crystals.

## Conceptual designs of dedicated/irregular PET scanners

**Fig. 1:**
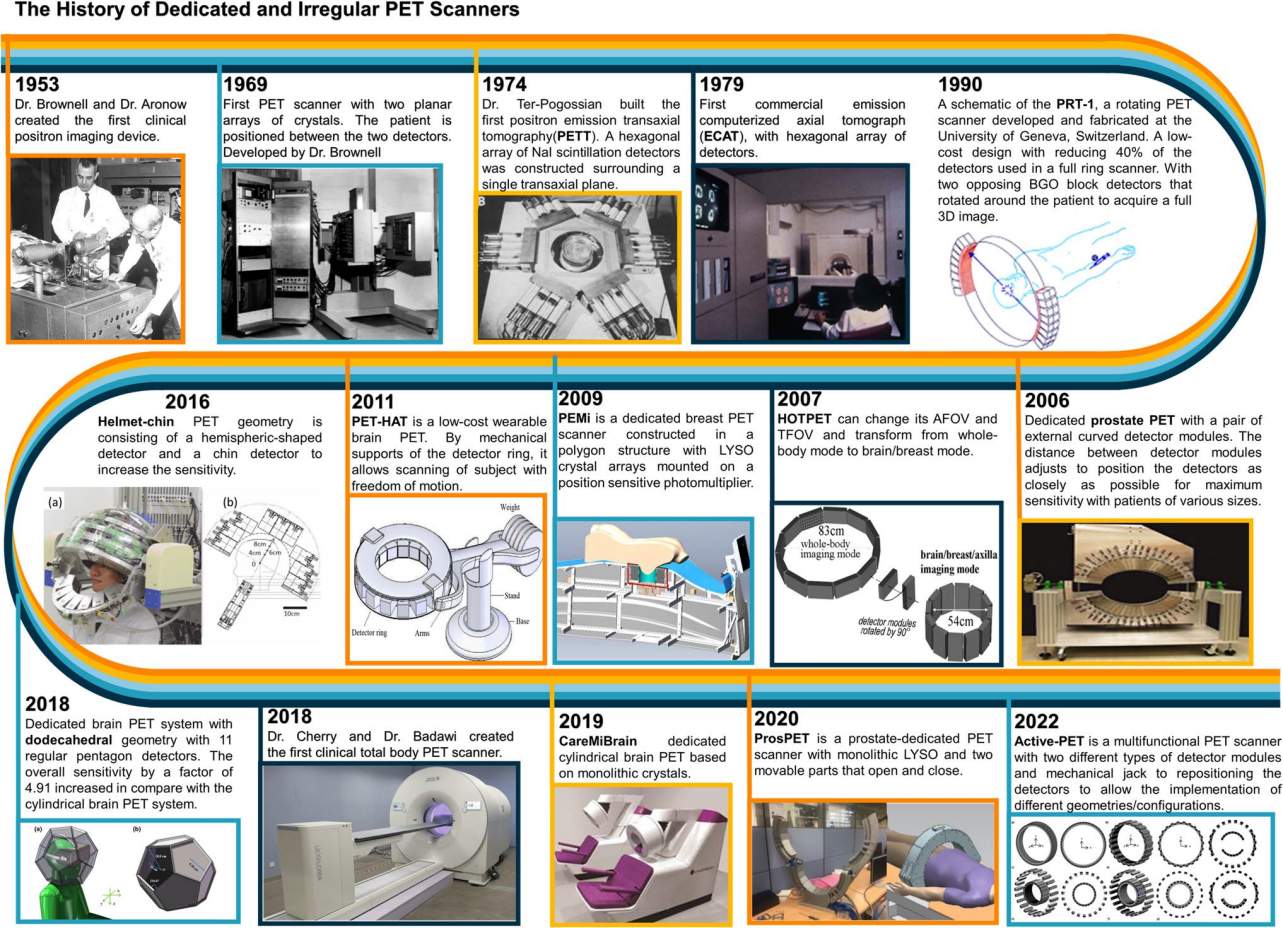
Overview of dedicated/irregular PET scanner geometries over the last 70 years. Courtesy of various sources, Refs. [5, 55, 58, 68, 69, 118–125]. ^©^IEEE. Reprinted, with permission, from Refs. [68, 121–123]. ^©^RSNA, Reprinted, with permission, from Ref. [119]. ^©^SNMMI Reprinted, with permission, from Ref. 120. ^©^IOP Publishing Ltd. with permission, from [5, 55]. ^©^John Wiley & Sons. Reprinted, with permission, from Ref. [58].

Multi-purpose or conventional PET scanners are intended for almost all clinical applications, including static whole-body, dynamic, brain, cardiac, prostate, and breast scans, as well as absorbed dose verification in heavy-ion radiation therapy [44, 45]. In Tables 2, 3, 4, 5, we listed and categorized dedicated/irregular PET scanners for brain, breast, prostate, and cardiac imaging, respectively. The design and performance parameters, such as spatial resolution, sensitivity, type of scintillator, and geometrical configuration, were listed to enable a quick comparison between the models. One column provides the technical details to provide the hidden aspects of the scanner design. Figure 1 depicts a short history of dedicated/irregular PET scanners development from the first dual-head PET scanner designed and developed in 1953 to the most technically complex and expensive total-body PET scanner. This figure covers a range of different geometrical designs from the dual-panel to dodecahedral geometry and moveable gantries with adjustable detectors.

**Table 2:** Summary of dedicated/irregular brain PET scanners

Name	Year	SR (mm)	Peak NECR (kcps)		Sensitivity	AFOV (mm)		TFOV (mm)	Scintillator		Sensor		Crystal size (mm^3^)	Geometry		Details
G-PET [134]	2003	4.2	60		4.79%	256		300	GSO		PMT		4 × 4 × 10	Cylindrical		_
jPET-D4 [135]	2006	3.1	82		19.3 cps/kBq	312		260	4-layer GSO		PS-PMT		2.9 × 2.9 × 7.5	Cylindrical		Four-layered DOI detectors
ECAT HRRT [136]	2007	2.6	45		2.5%	230		320	2-layer LSO/LYSO		PMT		2 × 2 × 10 and 10	Cylindrical		_
HOTPET [117] (brain mode)	2007	2.7	_		9.20%	210		530	BGO		PMT		2.68 × 2.68 × 18	Cylindrical		Axial and transaxial FOV change mechanically Can be transformed from whole-body mode to brain/breast mode
SBPET [137]	2009	2.7	8		1.14%	250		_	Liquid Xenon		_		32 × 50 × 100	Spherical		Spherical brain PET system with liquid xenon as scintillator
PET-Hat [138]	2011	4	0.82		0.72%	48		180	2-layer GSO		PF-PMT		4.9 × 4.9 × 7 and 8	Cylindrical		Moveable ring allowing subject freedom of motion Scanning can be performed in sitting posture The subject can move freely during PET data acquisition
Brain insert [139]	2012	1.8	30.7		7.2%	191		320	LSO		APD		2.5 × 2.5 × 20	Cylindrical		Insert inside the MRI
GAPD-PET [140]	2013	3.1	43.3		0.80%	60		390	LYSO		GAPD		3 × 3 × 20	Cylindrical		_
Rainbow VHD [141]	2013	1.4	_		_	119		300	LYSO:Ce		PMT		2.88 × 2.88 × 18	Cylindrical		_
NeuroPET [59]	2016	3.2	22.7		11.6 cps/kBq	220		250	LYSO:Ce		SiPM		2.3 × 2.3 × 10	Cylindrical		_
HelmetPET [142]	2016	2.8			0.70%	48		185	LYSO:Ce		MPPC		1.5 × 1.5 × 10	Cylindrical		3 kg in weight A ring with exterior weight support and an interior mechanism that could be fitted to the head
Neuro-PET [143]	2016	3.1	43.3		0.80%	60			LYSO		SiPM		3 × 3 × 20	Cylindrical		_
CerePET/πPET [144]	2016	2.1			_	85		220	LYSO		PMT		2.0 × 2.0 × 13.0	Cylindrical		_
Wearable brain PET (BET)* [145]	2016	1.2	_		_	_		_	LSO		_		1 × 1 × 3	Cylindrical		Lightweight and low-cost wearable helmet-shaped Brain PET Based on thin-film digital Geiger Avalanche Photodiodes Subject moves and acts freely and responding to environment
BrainPET-DOI [146]	2017	1.8	44.7		21.4 cps/kBq	201		330	4-layer LYSO		MPPC		1.2 × 1.2 × 3, 4, 5 and 8	Cylindrical		Four-layer DOI detector
MindView [147]	2017	1	_		2,7%	154		220	LYSO		SiPM		50 × 50 × 20	Cylindrical		PET/MRI Brain PET Insert
RF-penetrable PET insert [148]	2018	_			1.70%	280		320	LYSO		SiPM		3.2 × 3.2 × 20	Cylindrical		Radiofrequency field-penetrable PET insert for simultaneous PET/MRI
Dodecahedral scanner* [58]	2018	1.98	_		6.15%	_		_	LYSO		_		2 × 2 × 20	Dodecahedral		_
Helmet-chin PET [56]	2019	3			4%	~ 253		~ 253	4-layer GSO Zr-doped		PMT		2.8 × 2.8 × 7.5	Hemispheric		Include 3 types on add-on detectors: chin detector, neck detector, or ear detectors
CareMiBrain [125]	2019	2.34	49		11 cps/kBq	154		240	monolithic LYSO		SiPM		50 × 50 × 12	Cylindrical		Monolithic LYSO crystals
SAVANT [149]	2019	1.3	13		3.50%	235			LYSO:Ce		APD		1.12 × 1.12 × 12	Cylindrical		The basic detector consists of a dual-layer Phoswich array made of LGSO and LYSO scintillators
UHR* [149]	2019	1.3	16.4		3.40%	~ 235		271	LYSO		APD		1.12 × 1.1 2 × 12	Cylindrical		_
MINDView [150]	2019	1.7	_		7%	154		240	monolithic LYSO		SiPM		50 mm × 50 mm × 20 mm	Cylindrical		Hybrid molecular and anatomical imaging devices Brain PET insert, within a 3 T MRI
BPET [151]	2020	4	~ 2.4		2.9 cps/kBq	128		~ 242	LYSO		SiPM		4.1 mm × 4.1 mm × 10 mm	Cylindrical		_
Brain PET [152]	2020	~ 4	63.1		~ 1.5%	~ 230		~ 236	LFS		MPPC		4.14 × 4.14 × 10	Hemispheric		Include a hemispherical part and a neck part
NeuroEXPLORER [153]	2021	1.6	_		_	500		_	LYSO		_		_	Cylindrical		High sensitivity by increasing the coincidence acceptance angle, and high TOF resolution Good spatial resolution by reduction of detector elements size, DOI readout, and corrections for inter-crystal scatter Continuous motion tracking and correction
TOF brain PET [80]	2022	2.7	38.0		22.4 cps/(Bq/mL)	360		230	lutetium fine silicate (LFS)		MPPC		3.14 × 3.14 × 20	Cylindrical		Motion correction using system-based optical motion tracking Into the brain-dedicated TOF-PET scanner
TOF-DOI Prism-PET [82]	2022	1.53	_		1.20%	25.5			LYSO		SiPM		1.5 × 1.5 × 20	Decagon/Oval		SiPM pixels on one end and to a prismatoid light guide array on the opposite end
SIAT bPET^157^	2022	1.1	_		14.30%	329		240	LYSO		SiPM		1.4 mm × 1.4 mm × 20	Cylindrical		Dual-modality PET/MRI MRI compatible human brain PET insert
4D-PET [155]	2022	1.6	_		16.20%	~ 200		~ 280	semi-monolithic		SiPM		20 × 1.6 × 25.7	Cylindrical		4D-PET with a detector design based on semi-monolithic crystal Includes photon DOI measurement
VRAIN [156]	2022	2.2	144	25 kcps/MBq			224	224	lutetium fine silicate (LFS)	SiPM		4.1 × 4.1 × 10		Hemispherical	Includes a hemispherical part, and a half-ring behind the neck to cover the whole cerebellum The gantry can be tilted so as to align its axis with the orbitomeatal line	
TRIMAGE*, [157]	2022	1.9	129.9	7.61%			164	260	LYSO:Ce	SiPM		3.3 × 3.3 × 8 & 12		Cylindrical	Brain-dedicated PET/MRI/EEG	
BresTome^™^ (dedicated brain and breast PET) [158]	2022	2.5	35.2	7.18 cps/kBq			162	_	LGSO	SiPM		2.1 × 2.1 × 15		Cylindrical	Dedicated brain and breast PET system designed to switch between head scan and breast mode positions	
HNC PET [159]	2022	_	_	_			_	_	CZT	_		4 × 4 × 0.5		Dual panel	Two-panel head-and-neck dedicated PET based on CZT detectors The total system weight is less than 180 kg	
Voxel helmet brain PET [159]	2023	1.02	104.6	8 cps/kBq			154	133	CdTe	_		1 × 1 × 2		Cylindrical	Seamless geometry based on trapezoidal-shaped modules Equipped with semiconductor CdTe detectors	
Active PET brain mode* (small gantry) [5]	2022	2.3	135	15.98 kcps/MBq			218	350	LYSO	SiPM		2 × 2 × 10 4 × 4 × 20		Cylindrical	Multifunctional PET scanner consisting of two different types of detectors (thick and thin) Includes mechanical arms for repositioning of the detectors to produce various geometries/configurations	

*SR* spatial resolution, *AFOV* axial FOV, *TFOV* transaxial FOV

*Represents simulation studies

**Table 3:** List of dedicated/irregular breast PET scanners

Name	Year	SR (mm)	Peak NECR (kcps)	Sensitivity	AFOV (mm)	TFOV (mm)	Scintillator	Sensor	Crystal size (mm^3^)	Geometry	Details
BPET/CT [75]	2022	_	_	_	_	_	LYSO	PSPMT	2 × 2 × 15	Dual panel	PET component of the system consists of a rotating pair of 96 × 72 arrays of scintillator elements
Total-breastPET* [160]	2021	_	_	60.96 cps/kBq	_	_	LSO	_	3.2 × 3.2 × 20 1.6 × 1.6 × 6	Stadium shape ring	A ‘stadium’ (a rectangle with two semi-circles on opposite sides) shaped ring that includes both breasts, mediastinum and axilla
BPET-DBT [161]	2021	2	_	_	_	_	LYSO	_	1.5 × 1.5x15	Dual panel	TOF-capable breast PET scanner integrated with a digital breast tomosynthesis unit in a common gantry to provide co-registered PET-DBT images
PEM [162]	2002	4	_	0.07%(10°) 1.35%(40°)	150	200	LGSO	PSPMT	3 × 3 × 10	Dual panel	Non-fully tomographic imaging system
Dual round-edge detector [163]	2021	_	_	_	_	_	GFAG	SiPM	1.45 × 1.45 × 15	Dual panel	Dual round-edge detector arrangement, in which the detector blocks at both edge positions were tilted toward the center of the FOV
DH-Mammo PET [164]	2022	2.60	162.6	3.37%	120	216	LYSO	SiPM	1.89 × 1.89 × 13.00	Dual panel	Simultaneous positron emission tomography-Optical (OPET) breast imaging dual-head PET
DP-PET [77]	2021	2.5	_	3.60%	100	160	LYSO	SiPM	15.5 × 2.76 × 2.76	Dual panel	MR-compatible portable PET insert prototype Acquires simultaneous PET/MR imaging
BiPlanar Breast PET [165]	2020	1.5	319	_	_	_	_	_	_	_	Includes two movable paddles that can be placed in different Configurations to allow imaging of the breast and pectoral wall
EstatiraPET [94]	2020	2	21.8	1.42%	50	190	LYSO	SiPM	2 × 2 × 10	Cylindrical	_
PEM-FLEXSoloII [166]	2009	2.4	_	18%	164	240	LYSO	PMT	2 × 2 × 13	Dual panel	Non-fully tomographic imaging system
PEMsystem [167]	2010	1.2	42	11.50%	120	200	LYSO	PMT	1.5 × 1.5 × 10	Dual panel	Non-fully tomographic imaging system
C-shaped breast PET [168]	2009	0.7 1.2	~ 180	6.90%	105	216	LGSO	PMT	1.44 × 1.44 × 4.5	C-shaped	“C” shape configuration allows positioning around the breast Effectively increasing both resolution and sensitivity
PET [169]	2006	1.9	13.5	5%	50	82	LSO	PMT + SiPD,DOI	3 × 3 × 10	Rectangular	Four planar detectors covering the breast The rectangular arrangement using thick crystals enhances the sensitivity The parallax error is corrected by measuring DOI
ClearPEM [170]	2011	1.3	_	4.30%	145	165	LSO	PMT	3 × 3 × 20	Dual panel geometry	_
Dual panel PET/CT [171]	2009	2.7	19.3	1.46%	119	119	LSO	PMT	3 × 3 × 20	Dual panel geometry	The PET heads rotate in step and shoot mode The rotational steps were acquired over 180◦
BreastPET insert [172]	2009	2	_	_	18	100	LYSO	APD	2.2 × 2.2 × 15	Cylindrical	MR-compatible
Dedicated Breast PET [173]	2014	1.6	373.8	11.20%	155.5	183	LGSO	PMT	1.44 × 1.44 × 4.5	Cylindrical	_
DbPET2.1/CT [174]	2015	1.6	_	0.50%	50	175	LYSO	PMT	1.27 × 1.27 × 20	Dual curved panel geometry	Two curved heads in coincidence, spanning exactly 90° Vertical stages are used to position the PET curved head close to the chest wall and to cover the breast The whole PET/CT gantry rotates to acquire fully tomographic data
PEMi [174]	2015	1.5	110	6.88%	128	110	LYSO	PMT	1.9 × 1.9 × 15	Polygon structure	_
MAMMI-PET [175]	2016	2	125	2.00%	40	170	LYSO, Monolithic	PMT	40 × 40 × 10	Dodecagon Shape	The patient lies down in prone position during the scan This position enables better tumor delineation, differentiation, and localization than in supine position, A vertical elevator move the entire ring detector in a step and shoot mode to increase the axial FOV
PET/X [176]	2017	_	_	_	160	240	LYSO	SiPM	2 × 2 × 10	_	_
PEM/PET/CT [177]	2018	2.2	24.6	1.36%	150	150	LYSO	PMT	2 × 2 × 15	Dual panel	_
Circular shape breast PET [178]	2018	2.1	26	2%	50	260	LGSO	SiPM	1.5 × 1.9 × 15	Cylindrical	_
PET* (Ring mode, 19 mm crystal thickness) [179]	1997	~ 4	_	4.89 kCts/Ci	~ 200	~ 172	NaI(Tl)	_	_	Cylindrical	The first design is a cylindrical scanner surrounding the breast The second design consists of two planar detectors placed on opposite sides of the breast
HOTPET (In breast mode configuration) [117]	2007	2.7	_	9.20%	210	540	BGO	PMT	2.68 × 2.68 × 18	Cylindrical	Axial and transaxial FOV change mechanically Can be transformed from whole-body mode to brain/breast mode
PEMI [180]	2000	2.8	_	3%	72	72	BGO	PMT	1.9 × 1.9 × 6.5	Dual panel	_
Pisa [181]	2011	_	_	_	100	100	LYSO	PSPMT	1.9 × 1.9 × 16	Dual panel	_
maxPET [182]	2001	4	_	0.57%	150	150	LSO	PSPMT	3 × 3 × 20	Dual panel	_
Stanford Breast PET [183]	2016	0.9	_	_	100	160	LYSO	PSAPD	0.9 × 0.9 × 1	Dual panel	_
Active PET breast mode* [5]	2022	2.9	40	6.82 kcps/MBq	218	350	LYSO	SiPM	2 × 2 × 10 4 × 4 × 20	Oval shape	_
Radialis [184]	2022	2.3	17.8	3.5%	170	220	LYSO	SiPM	2.3 × 2.3 × 13	Dual panel	Multi-organ PET with a movable gantry

*SR* spatial resolution, *AFOV* axial FOV, *TFOV* transaxial FOV

*Represents simulation studies

**Table 4:** List of dedicated/irregular prostate PET scanners

Name	Year	SR (mm)	Peak NECR (kcps)	Sensitivity	AFOV (mm)	TFOV (mm)	Scintillator	Sensor	Crystal size (mm^3^)	Geometry	Details
Compact PET* [185]	2001	4	_	_	_	_	BGO	PMT	4.5 × 4.5 × 30	Dual-curved panel	The lower detector module is fixed below the patient bed. The top module is adjustable vertically
Planar PET [186]	2004	~ 3	_	_	_	_	LGSO	PS-PMT	3 × 3 × 10	Dual panel	_
Dual-Modality PET/Ultrasound [68]	2006	4	_	946 cps/µCi (2.6%)	_	_	BGO	PMT	4.4 × 4.1 × 30	Dual panel	Includes a pair of curved detector modules The two modules form an incomplete elliptical ring which reduces the distance between the detectors and patient The distance between detector modules and patient is adjustable
Intra-operative PET imaging Probe* [187]	2007	_	_	_			LSO and BGO	PMT	2 × 2 × 3 5 × 5 × 30	Unusual	Includes a curved detector placed back of the patient and a small PET imaging probe The coincidence events between the curved detector and the small imaging probe are collected The PET imaging probe is equipped with a position tracker which enables the clinicians to survey suspicious regions by moving the probe during the imaging process
Internal PET probe* [187]	2007	1	_	_	_	_	LSO and BGO	APD	1 × 1 × 3 4.2 × 4.2 × 30	Cylindrical with internal probe	The internal detector probe operates in coincidence with a ring of detectors
Mobile prostate PET [188]	2010	_	_	_	_	_	_	_	_	Dual panel	_
Stereotactic PET [189]	2011	1	_	_	100		LYSO	MPPCs	1.5 × 1.5 × 10 4.2 × 4.2 × 30	Dual panel geometry with internal probe	Includes an endorectal PET probe and two PET panel imaging modules Provides two instant reconstruction (aluminography) and simultaneous stereotactic views of the prostate region
TOPEM [61]	2013	1.5	_	_	_	_	LYSO	SiPM	25 × 50 × 13	Carved detector with internal probe	An endorectal PET-TOF MRI probe
EndoTOFPET-US [190]	2015	1	_	_	_	_	LYSO	SiPM	0.71 × 0.71 × 15	_	Internal probe can be in coincidence with external plane A multimodal device for ultrasound endoscopy and PET Using TOF information In endoscopic procedure, the PET detector is mounted on the transrectal ultrasound endoscope
PROSPET* [113]	2019	_	_	_	_	_	Monolithic LYSO	SiPM	5 × 50 × 15	Dual panel	Open geometries include TOF
ProsPET [69]	2020	2	16	1.46%	46	300	Monolithic LYSO	SiPM	50 × 50 × 15	Cylindrical	The system has two movable parts that open and close from left to right

*SR* spatial resolution, *AFOV* axial FOV, *TFOV* transaxial FOV

*Represents simulation studies

**Table 5:** Summary of dedicated/irregular cardiac PET scanners

Name	Year	SR (mm)	Peak NECR (kcps)	Sensitivity	AFOV (mm)	TFOV (mm)	Scintillator	Sensor	Crystal size (mm^3^)	Geometry	Details
AttriusPET [191]	2010	5.8	_	_	124	166	BGO	PMTs	8.5 × 9.8 × 30	cylindrical	Detectors operate in 2D mode Increase sampling using ‘wobble’ technology
Cardiac TOF-PET System* [192]	2020	3.82	_	3.87 cps/kBq	280	280	LYSO	_	50 × 50 × 15	4 Planar detectors	Asymmetric open geometry Two detector panels back and front of the chest. Two detector panels left and right of the patient
Cardiac TOF-PET System* [192]	2020	4.01	_	2.17 cps/kBq	280	280	LYSO	_	50 × 50 × 15	Arc and planar detectors	Arc of detectors back of the patient. Three planar detectors front left and right
Compact ellipse cardiac TOF-PET* [193]	2021	2.4	425	16.60%	210	~ 400	LYSO	SiPM	4.0 × 4.0 × 20	Elliptical	Elliptical geometry with 30 cm and 40 cm short and long diameters
Compact D-shape cardiac TOF-PET* [193]	2021	2.5	422	14.50%	210	~ 400	LYSO	SiPM	4.0 × 4.0 × 20	D-shape	D-shape arrangement of detectors, Flat part is back, curved part is in front

*SR* spatial resolution, *AFOV* axial FOV, *TFOV* transaxial FOV

*Represents simulation studies

**Fig. 2:**
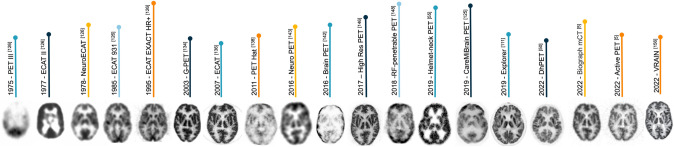
Brain PET images of humans or 3D Hoffman brain phantom acquired by different simulated or physical dedicated, conventional, and irregular PET scanners. Courtesy of various sources, Refs. [5, 50, 55, 111, 125, 126, 134, 136, 138, 142, 143, 146, 148, 156]^.^ The images related to reference [126] were taken from https://www.cerebromente.org.br/n01/pet/pet_hist.htm. ^©^IEEE. Reprinted, with permission, from Refs. [138, 148], ^©^SNMMI Reprinted, with permission, from Refs. [50, 111, 134], ^©^IOP Publishing Ltd. with permission, from Refs. [5, 55, 136, 146]

In Fig. 2, we illustrated the improvement in image quality through depicting the 3D brain phantom from 1975 to 2022. This anecdotal illustration provides a sense of images generated by these systems as the data acquisition and reconstruction protocols were different. Yet, one can observe that scanners with small geometrical coverage (e.g., Helmet and PET-Hat) lead to quality degradation and provide less anatomical information. The insert PET scanners, like RF-penetrable, also generated blurred images, which can be caused by inaccurate attenuation correction.

The key factors in the conventional PET scanners are robustness, reproducibility, and accuracy of quantitative imaging to guarantee/ensure a dependable/reliable examination for the different applications (screening, diagnosis, response to treatment, and follow-up) considering the high throughput of patients in clinical setting. To fulfill the clinical requirements, conventional/multi-purpose PET scanners should provide relatively high sensitivity, moderate spatial resolution, at reasonable cost, and last but not least accurate/reproducible image quantification (since quantitative imaging is crucial in most clinical indications). The compromise/trade-off among these factors is considered in the design of conventional PET scanners, wherein the equipment used for simultaneous or sequential transmission or anatomical imaging is well considered, since it plays a significant role in quantitative PET and synergistic functional–structural imaging [46].

This trade-off would be highly skewed in dedicated PET scanners, since one of these key factors may have central importance in organ- and/or application-specific PET scanners [47]. For instance, in dynamic whole-body PET imaging, sensitivity is the key factor for low-noise estimation of time-activity curves (or parametric maps). Hence, the tendency would be toward extended FOV PET scanners (through adding more detector rings) at the cost of increased product price [48] or having axial gaps and covering a larger AFOV with the same number of detectors as demonstrated on the PennPET Explorer [49]. On the other hand, in brain PET imaging, a higher spatial resolution would be appreciated to register underlying signals from fine brain structures and neuro-connections. Hence, the tendency is toward exploiting/designing high-resolution PET detectors (finely pixelated, thin monolithic crystals, DOI capability, and advanced electronic read-outs) [50].

Organ-specific dedicated PET scanners are often designed to accommodate the target organ while maximizing the sensitivity and SNR. Nonetheless, the compact design of such scanners with small gantry aperture potentially increases parallax errors, thus degrading the spatial resolution uniformity. To alleviate this issue, detectors with discrete or continuous DOI capability are frequently considered (see [51, 52] for a review on various DOI techniques). Careful detector modules’ geometrical optimization has been reported in the literature to maximize performance from various standpoints. The use of several multi-layer LYSO crystal arrangements to improve spatial resolution uniformity and count-rate performance of a compact MR-compatible PET insert were reported [53].

In this review, we attempted to cover all PET scanners belonging to the following categories:

- Organ-specific dedicated PET scanners (e.g., brain, breast, prostate, etc.).
- PET scanners with non-cylindrical “asymmetrical” geometries (e.g., planar, partial-ring, oval shape, spherical/hat shape, etc.).
- Cylindrical geometries with moveable detectors or gantry.
- Any kind of cylindrical PET scanners bearing some novelty in detector modules’ conceptual design and acquisition techniques.

Some conceptual designs never materialized in real systems demonstrating their potential in clinical setting. Yet, they are briefly discussed in this review for the sake of completeness.

## Challenges of PET image reconstruction algorithms for multifunctional PET scanners

Fulfilling the desired high performance of dedicated PET scanners requires the application of proper correction and calibration algorithms. Geometrical symmetries in PET scanners are often used in the calculation of the geometrical components of normalization factors [54]. Nonetheless, organ-specific dedicated PET scanners are designed to maximize the sensitivity when imaging the target organ, which often calls for a geometrically asymmetrical scanner. For instance, a peak sensitivity of more than 10% was achieved by the helmet PET scanner with an added row of detectors along the chin [55, 56]. The proposed helmet-chin scanner achieved 40% higher peak noise equivalent count rate (NECR) compared to a cylindrical PET scanner with the same number of detectors [57]. A similar dodecahedral design benefiting from an almost 4π coverage was suggested by [58]. While such designs boost detection efficiency of an organ-dedicated PET scanner, their asymmetrical geometry adds complexity to the normalization and correction of PET data.

Regarding PET data correction, some organ-specific dedicated PET scanners use concurrently acquired anatomical images from CT or MRI scanner. For instance, NeuroPET [59] includes a CT scanner. Likewise, MR-compatible PET inserts [60, 61] can benefit from the anatomical MR images for both anatomical localization and attenuation and scatter correction (though converting MR images to attenuation maps is another source of complexity). Anatomical MR images have also been used to estimate motion vectors to correct for patient head motion in PET/MR neuroimaging applications [62]. However, the absence of an anatomical imaging modality on most organ-dedicated scanners brings new challenges to PET attenuation and scatter correction which might consequently compromise PET’s quantitative accuracy. In the case of brain imaging, atlas-based u-maps generation was extensively studied [63, 64]. Nonetheless, such approaches are increasingly more challenging and less reliable when imaging other organs. Therefore, attenuation and scatter corrections are sometimes ignored on such scanners [65, 66] or alternative innovative approaches are sought. In addition, detector gain adjustment is a critical consideration that can affect peak location, scatter contribution, and consequently overall image quality in all PET scanners and more specifically on MR-compatible PET inserts [67].

A prostate PET scanner was designed [68] with the unique feature that it can be tilted to minimize photon attenuation effects [69]. Existing CT images from a separate scan can be co-registered to PET images to perform attenuation correction on dedicated prostate PET scanners. Another challenge in the reconstruction of organ-specific scanners is that often a large part of the data might be missing due to the inevitable detector gaps. These can be handled through interpolation, forward projection of an initial image estimate, or directly in the projection domain using deep learning (DL)-based approaches [70–73].

Table 6 summarizes the challenges and innovations in image reconstruction for a selection of dedicated/irregular PET scanners. In this table, we categorized image reconstruction methods for each geometrical configuration (Cylindrical, Cylindrical with removed/added detector modules, Flat-Panel, Spherical/Pseudo-spherical, and Irregular configuration) and target organ. The potential challenges and drawbacks for each configuration as well as the strategies for addressing them were listed. Furthermore, the calibration and correction method used in these scanners were briefly mentioned.

**Table 6:** Summary of the unique challenges and features of some dedicated/irregular PET scanners from a reconstruction point-of-view

Geometry	Scanner name	Target organ	Reported potential challenges for reconstruction	Added features to address the challenges	Reported data calibration/correction methods
Cylindrical	ECAT HRRT [194]	Brain	1. Parallax error 2. Image artifacts due to 1.7-cm gaps between panel detectors	1. 2-layer DOI detector 2. Application of 3D iterative reconstruction or an initial reconstruction followed by forward projecting the image in the gaps into the sinograms to create the missing data for FORE—2D-OSEM	Random correction using delayed coincidence window Attenuation correction using a ^137^Cs transmission point source
	G-PET [134]	Brain	1. Conversion of u-values from 662 keV (^137^Cs) to 511 keV 2. Emission contamination in transmission energy window	1. Approximation by linear scaling 2. Subtraction of a mock scan without ^137^Cs source from the transmission data or contamination estimation from singles rate	Normalization Random subtraction using a delayed coincidence window Attenuation correction based on the singles method using a ^137^Cs transmission point source Scatter correction using a background tail-fitting algorithm like or a model-based scatter correction
	jPET-D4 [135, 195]		1. Parallax error 2. Sampling errors due to irregular sampling of the DOI detectors 3. Reconstruction computation cost due to 4-layer DOI detectors 4. Dark bands on some regions of images of uniform background due to normalization mismatch	1. 4-layer DOI detector 2. Using a new histogramming method [Hagiwara 2003] based on detector response functions 3. Application of DOI compression before reconstruction 4. Not reported	Normalization using rotation of 3 ^68^Ge–^68^ Ga line sources Random correction using delayed coincidence window
	HOTPET (in brain/breast mode) [117]	Brain or Breast or Axilla	1. Parallax error 2. Potential image artifacts due to limited spatial sampling	1. An iterative DOI reduction technique in the sinogram domain with model-based PSF using Monte Carlo 2. 30° rotation of gantry in 1°-steps	Random, attenuation, geometric and detector pair efficiency corrections: methods not reported No scatter correction
	PET-Hat [138]	Brain	1. Parallax error 2. Low image quality of Hofmann brain phantom due to the system low sensitivity, small axial FOV (44 mm), short scintillators depth, low NECR, and use of FBP reconstruction	1. 2-layer DOI detector 2. Lowering energy level or time window may improve and applying an iterative image reconstruction may/will improve the image quality	Normalization Random correction using subtraction of delayed window Attenuation correction using analytical correction Scatter correction based on single value subtraction
	Rainbow VHD [141]	Brain and head and neck	1. Motion-induced image blurring	1. Fixed patient table while moving the detector ring	Attenuation correction using external CT/MR image or estimated attenuation based on obtained boundary from the PET image
	A Dedicated breast PET scanner [173]	Breast	1. Parallax error 2. High noise with the enhanced-resolution mode reconstruction	1. 4-layer DOI detector 2. Propagation of noise from projection data to the final image could be controlled by adjusting the dynamic relaxation parameter of 3D list-mode DRAMA	Attenuation correction using external CT image Scatter correction using convolution subtraction method with kernels obtained by background tail-fitting
	Helmet_PET [142]		1. Low resolution of patient images due to prototype nature of the study 2. Non-ideal normalization method 3. Need to blur images due to noise presence owing to lack of scatter and random corrections 4. Remaining portions of the brain cortex out of FOV 5. Loss of spatial resolution toward the FOV edge	1. Not reported in the study 2. Sophisticated normalization with modeling of detector response in the next generation of the scanner 3. Implementation of basic corrections in the next generations 4. Increasing the scanner diameter in the next generations 5. DOI correction could mitigate it partially if implemented	Normalization Attenuation correction assuming the whole volume inside the imager is water No random and scatter corrections in the study
	Central-Research-Laboratory Brain PET [146]	Brain	1. Parallax error 2. Reconstruction computation cost	1. 4-layer MPPC DOI detectors 2. Introduction of high-resolution and high-sensitivity modes using different crystal segments	Component-based normalization (CBN) Dead-time correction based on an empirical relation between the total single count rate and the true coincidence count rate Random correction by subtracting delayed coincidence events Emission segmented attenuation correction (E-SAC) attenuation correction using segmented attenuation map generated from emission data Scatter correction using Single-scatter simulation
	NeuroPET/CT [59]	Brain	1. Parallax error	1. 2-layer DOI detectors (DOI not applied in the study)	Normalization Dead-time correction using a paralyzable dead-time model at the block level based on block singles rates Randoms correction using subtraction of smoothed delayed data Integrated-CT-based attenuation correction using bilinear scaling to convert CT images to µ-values Scatter correction using single-scatter simulation Decay correction per-frame
	Neuro-PET [143]	Brain	1. Parallax error 2. Low sensitivity and peak NECR due to short axial extent (60 mm) and system dead-time	1. Applying iterative reconstruction using system matrix in future works 2. Extending axial FOV, better shielding of out-of-FOV activity, and optimization of acquisition signal processing in future works	
	Siemens PET Insert for MR [139]	Brain (PET insert for MR)	1. Additional scatter and attenuation from RF coil in the PET FOV 2. MR-based attenuation correction	1. Scatter and attenuation correction for RF coil 2. Pseudo-CT image generation from MR images [Hofmann 2008]	Normalization using a count-rate-dependent method Partial pile-up rejection by the front-end firmware Dead-time correction using a global scaling factor in image space MRI-based attenuation correction Scatter correction
	MINDVIEW [147]	Brain (PET insert for MR)	1. Parallax error	1. Monolithic crystal with DOI determination	Random correction No attenuation correction Scatter correction
	RF-penetrable PET [148]	Brain (PET insert for MR)	1. Low SNR of images due to low sensitivity of 2.8-cm axial FOV 2. Artifactual hot regions in Hofmann phantom image (perhaps) due to using a cylindrical source for normalization and attenuation correction	1. Extending axial FOV or improving timing resolution to enable TOF acquisition can resolve the issue in future 2. Using an annulus source for normalization and scatter correction might mitigate this issue	Normalization Random correction by subtracting a delayed window No scatter correction
	CareMiBrain [125]	Brain	1. Parallax error	1. High-resolution (1 mm) DOI determination in the monolithic crystal	Direct normalization method Random correction using the singles rate method Attenuation correction by segmentation of the emission image Scatter correction based on the dual-energy window method
	BPET [151]	Brain	Not reported	Not reported	Random correction using singles method Attenuation correction using homogeneous u-maps estimated from segmentations of the emission data
	HIAS-29000 [80]	Brain	1. Parallax error 2. Image degradation from patient motion 3. 16-mm gap between 2 of the detector rings for placement of motion capture system	1. Accepted 2. Motion capture using an optical tracking system and motion correction 3. Will investigate the gap effect on clinical images in their future studies	Component-based normalization Random correction by subtracting delayed coincidence events Attenuation correction using segmentation of the emission image Scatter correction based on a single-scatter simulation method
	4D-PET [155]	Brain	1. Parallax error	1. 3D photon impact positioning in crystal-slab detectors using a neural network	Simulation study: no data correction
	Trimage [157]	Brain (PET/MR/EEG)	1. Parallax error	1. 2-layer DOI detector with staggered structure for better sampling of FOV	Simulation study Method for normalization and attenuation corrections: not reported Image space modeling of spatial resolution in reconstruction
	BresTome [158]	Brain and breast	1. Parallax error	1. No adverse effect was foreseen for clinical images using iterative reconstruction with TOF data	Random corrections using delayed events Count loss correction Attenuation correction with a modified maximum-likelihood attenuation correction factor [108] Scatter correction using single-scatter simulation
Cylindrical with removed/added detector modules	ProsPET [69]	Prostate	1. Imaging-guided prostate biopsy 2. Parallax error 3. Low sensitivity for prostate imaging 4. High random and scatter contribution	1. Tight detector ring around hip 2. DOI from energy signals of all channels 3. Tighten the PET inner diameter to 41 cm 4. Work in progress	Normalization using data from an annulus phantom Attenuation correction using CT images from a separate scanner or segmentation for phantoms
	Intra-operative PET probe [187]	Intra-operative	1. Low sensitivity 2. Flexible position of imaging probe	1. Probe can get very close to tumor 2. Use of a position tracking device	Simulation study Normalization through solid-angle calculations using vector notations
	C-Shaped PET [168]	Breast	1. Parallax error and sensitivity 2. Image artifacts due to limited angular coverage	1. 4-layer DOI detector 2. Work ongoing on optimizing a reconstruction algorithm	Not reported
	Active PET [5]	Multifunctional	1. Mechanical and electrical issues due to varying geometry 2. Varying sensitivity	1. Use of flat flexible cables, actuators and electro-optical fibers 2. Sensitivity map for each geometry found through GATE	Simulation study Normalization: geometrical components modeled by CASToR
	NeuroEXPLORER [196]	Brain	1. Sensitivity 2. Resolution and resolution uniformity 3. Low SNR 4. Head motion	1. Extended axial FOV brain (~ 50 cm) PET w/ potentially a partial detector ring to accommodate shoulders. Sensitivity of > 10-folder higher than HRRT 2. Use of 1.52 mm crystals w/DOI resolution of less than 4 mm 3. TOF resolution of < 250 ps 4. Use of a real-time stereovision system	Simulation study Attenuation correction using a clinical CT
Flat panel	PEM Flex Solo II [197]	Breast	1. Parallax error 2. Worse cross-plane spatial resolution compared to in-plane resolution due to limited angular coverage	1. Limiting LOR angular range to reduce DOI effects 2. Not reported	No corrections for randoms and scatters No attenuation correction
	Prostate PET w/ Transrectal tube [188]	Prostate	3. Low sensitivity due to small probe size 4. Slow response due to the use of probe during surgical operations	3. External detectors will create images with moderate sensitivity 4. Recon using fast LM-based methods	Not reported
	TOPEM [61]	Prostate	1. Sensitivity 2. Low SNR	1. Use of an endorectal probe 2. Potential addition of more external detector panels	Simulation study
	Stereotactic PET [189]	Prostate	1. Parallax 2. Spatial resolution	1. DOI (~ 1 mm FWHM) 2. Use of an endorectal PET probe and two PET panel modules	Motion tracking for the probe using a MicroBird EM tracking system
	HNC add-on PET [198]	Head and neck	1. Sensitivity	1. Add-on dedicated head-and-neck scanner to complement whole-body PET	Simulation study
	BPET-DBT [161]	Breast	1. Limited-angle image artifacts	1. TOF-capable detector—still additional blurring along y-axis	Not reported in detail—ongoing
	DP-PET (insert for MR) [77]	Breast	1. Worse spatial resolution along the axis perpendicular to the detector panels due to limited angular coverage	1. Not reported	Normalization using a plane source Random correction using a delayed window Attenuation correction using segmentation of MRI images (3D Dixon in-phase/out-phase imaging sequence) Scatter correction based on a single-scatter simulation with L1-norm tail-fitting
Spherical/Pseudo-spherical	SBPET [137]	Brain	1. Sensitivity	1. Spherical design and the use of thick liquid scintillators (liquid xenon)	Simulation study
	TOF-DOI Prism-PET [82]	Brain	1. Limited spatial resolution and parallax error	1. DOI-enabled highly pixelated crystals	
	Helmet and Helmet-chin [54, 57]	Brain	1. Parallax error 2. Sensitivity	1. 4-layer DOI detector 2. A helmet PET with an added series of 7 chin detectors	Simulation study Attenuation correction using a separate CT is suggested (not implemented)
	Polyhedron brain PET [58, 199]	Brain	1. Sensitivity 2. Retrofitting detectors to spherical surfaces	1. Maximize sensitivity by maximizing covered solid angle 2. Use of a polyhedron	Simulation study
	VRAIN [156]	Brain	1. Parallax error 2. Sensitivity 3. Low SNR	1. Limiting crystal length to only 10 mm 2. Hemispherical design w/ one-to-one couple LFS scintillators 3. Avoid compromising TOF by not using DOI	Normalization and time calibration using a hollow-dome phantom Randoms correction using a delayed window method Attenuation correction requires external CT or MR for attenuation maps
Irregular	Conceptual PET for prostate [68, 185]	Prostate	1. Parallax error 2. Sensitivity 3. Random and scatter events 4. Irregular and incomplete sampling due to detector side gaps	1. Angled detector modules toward the scanner center near the prostate; DOI capability reported as not necessary 2. Moving upper detector arc to reach maximum sensitivity 3. Extended interplane septa to reduce randoms and scatters 4. Nearly artifact-free images using iterative reconstruction	Simulation study Attenuation correction considering uniform attenuating media in the body
	PET for prostate [68, 185]	Prostate	1. Sensitivity	1. Adjustable detectors arranged in an elliptical shape	Randoms correction using a delayed window method Attenuation estimated for phantom
	LBNL PEM [169]	Breast	1. Parallax error 2. 64-fold increase in the number of LORs makes it inefficient to process the data in histogram format 3. Irregular radial and angular sampling in this rectangular geometry	1. 8-layer DOI detector 2. Development of a list-mode maximum-likelihood algorithm explicitly modeling the DOI and rectangular geometry	Normalization using a rectangular flood phantom Random correction using a delayed window No attenuation and scatter correction
	EndoTOFPET-US [190]	Prostate	1. Anato-functional imaging of prostate	1. PET head extension attached to an US transducer with an external PET detector plate	
	AGPET [200]	Adjustable—multi-purpose	1. Parallax error due to square-shape gantry 2. Sensitivity	1. DOI using DLO geometry and reflector pattern 2. Adjustable gantry to adapt FOV	Simulation study

## Summary and future trends

**Fig. 3:**
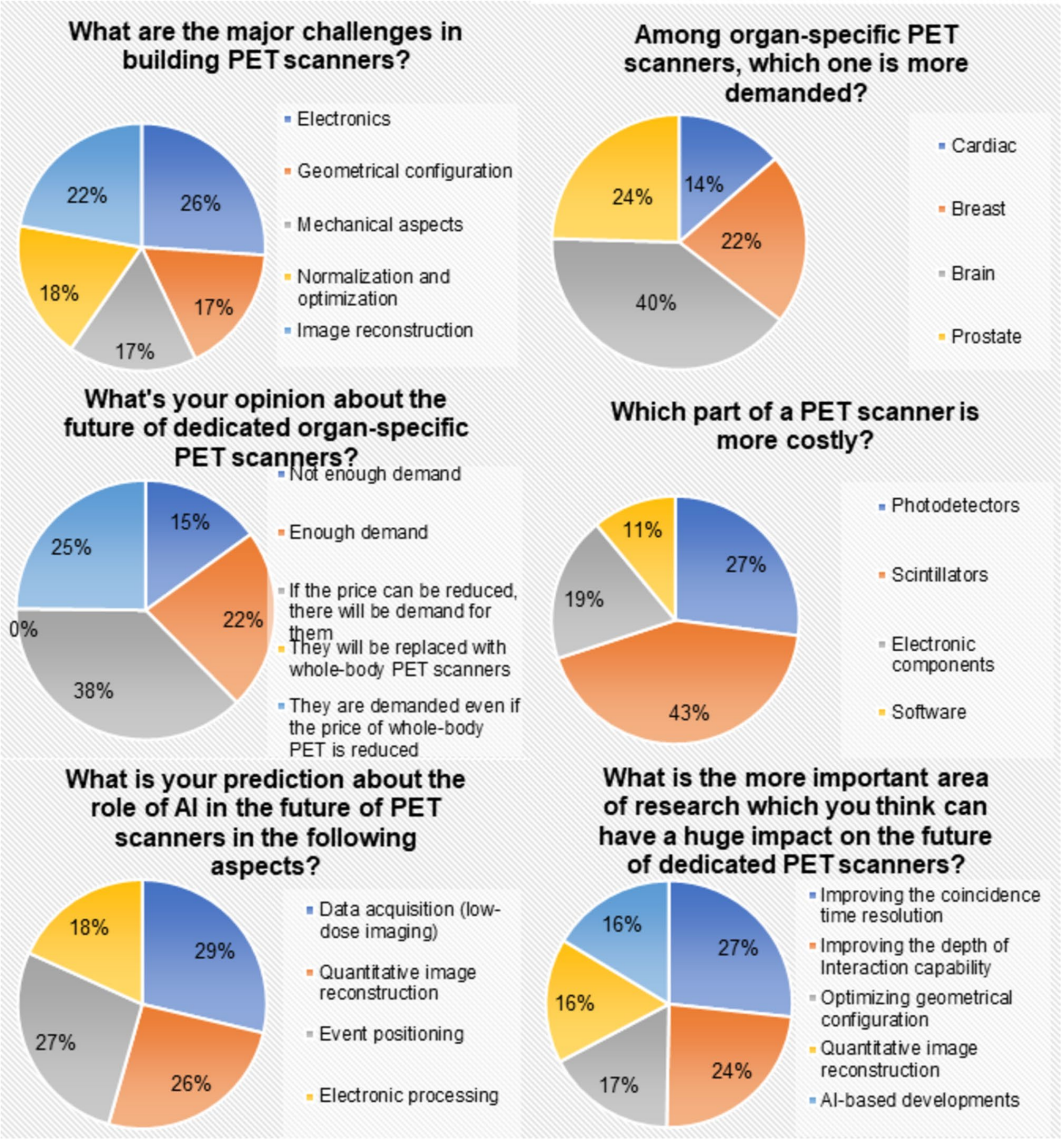
Outcome of a survey from 15 experts in the field of PET instrumentation who provided feedback on six main questions about dedicated PET scanners and future developments

To reflect the perspectives/opinions of experts in the field of PET instrumentation, a survey was designed for this review and sent to 15 PET scientists. This survey included six questions about the design and development of dedicated PET scanners and the future of this field of research. We asked experts in the field to give a score between 1 and 10 for less important to more important or less costly to high costly. For other questions, we also asked them to sort out the options. The response to the questionnaire was averaged and the answers are reported in Fig. 3. Based on this survey, we concluded that the major challenges in dedicated/irregular PET scanner fabrication lie in the optimization of electronics and image reconstruction methods which take the utmost of human resources, while the scintillators and photodetectors take the utmost of financial resources. The majority of the experts also felt that dedicated brain and prostate PET scanners have the highest request, and if the price can be reduced, there will be a large demand. Definitely, none claimed that they will replace whole-body PET scanners. The results also supported the argument that there is a large space for AI to play role in data acquisition, event positioning, and quantitative image reconstruction and that future research should focus more on improving the coincidence time resolution, depth of interaction, and optimal geometrical configurations.

The major motivation behind the design and manufacturing of organ-specific PET scanners is to reduce the cost of end products compared to conventional cylindrical multi-ring PET scanners without scarifying key image quality factors relevant in clinical applications. Yet, the aim of dedicated scanners is not to replace existing clinical whole-body PET systems. In this regard, many efforts have been spent toward the design and building of compact PET scanners dedicated for specific organs with remarkably decreased manufacturing costs by reducing the complexity of PET systems’ design (e.g., using flat panel detectors), reducing the number of detectors, and using cost-effective PET detectors, such as monolithic scintillation crystals [74–76]. Owing to high demand for brain, breast, and prostate PET scans, the majority of dedicated PET scanners were designed for the purpose of reducing overall public health costs and increasing the accessibility of PET scanners to remote areas and/or developing/underdeveloped countries [75, 77]. A 2-m-long total-body PET scanner with plastic scintillators, referred to as J-PET [7], is one example of attempts to reduce the cost of a total-body PET scanner. Plastic scintillators used in J-PET have a density of about 1 g/cm^3^ (whereas LSO and BGO have a density of 7.1 to 7.4 g/cm^3^, respectively) but can provide decent time resolution (about 220 ps CTR) at the cost of reduced sensitivity [7, 78]. To reduce fabrication cost, a number of groups considered rearranging and reducing the number of detectors while relying on DOI and TOF to compensate for the missing sections (see for instance Ref. [79]).

Since conventional PET scanners are normally capable of providing moderate spatial resolution, a major incentive for dedicated PET scanners is to achieve high spatial resolution of the desired structures, such as in brain imaging. The majority of high-resolution dedicated PET scanners are designed for brain imaging, wherein quantitative and high-resolution imaging of brain-specific radiotracers in small structures and neuro-connections is highly desirable [80].To this end, high-resolution pixelated detectors improved DOI and TOF capability, whereas high-speed electronic readout technologies are employed on dedicated brain PET scanners [81, 82]. Furthermore, owing to the small FOV required in brain PET imaging, high-sensitivity imaging could be easily achieved by covering the whole head area, as used on the helmet PET scanner [56].

In addition to achieving low-cost (for prevalent PET scans, such as prostate imaging) and high-resolution (for dedicated brain studies) PET imaging, the motivation behind designing dedicated PET scanners targets specific applications that cannot be accomplished with conventional PET scanners. Range verification in heavy-ion therapy (such as proton radiation therapy) plays a key role in accurate radiation treatment planning monitoring, wherein the identification of the Brag peak location is crucial to deliver the maximum radiation dose to the target volumes and spare healthy/normal tissues [83, 84]. Online (in-beam) PET imaging in heavy-ion radiation treatment requires an open gantry PET design for direct access of radiation beams to the patients [84]. A two-panel PET design is commonly considered for online PET imagers, where the patient could be accessed from two other sides. Due to the fact that the rate/probability of positron emission is not very high, these PET scanners should be equipped with high-sensitivity detectors to achieve acceptable SNR (sensitivity has higher priority than spatial resolution in this case) [85, 86]. Another interesting design, referred to as human-scale single-ring OpenPET system, providing an open space area by axially shifting the detectors to different sides in the axial direction, is suitable for online range verification in heavy-ion therapy [87].

In addition, simultaneous imaging of the target areas is crucial to achieve accurate whole-body dynamic and parametric PET imaging. This would also obviate the need for blood plasma sampling (input function) provided that the major body blood pools are covered in PET imaging [88]. In this regard, extended FOV or total-body PET systems gain attention for enabling fully parametric imaging as well as very low-dose or ultra-fast PET scans [49, 89]. The key factor in the design of such systems is the extensive coverage of the body at the cost of a dramatic increase in manufacturing expenses. To address this issue, extendable FOV PET scanners have been proposed/designed to reduce the number of required PET detectors (to reduce the overall manufacturing cost) while providing the required axial FOV. In these PET scanners, the detector rings are mounted on a mechanical mechanism allowing for an axial extension [90–92]. Furthermore, arterial blood sampling is crucial (regarded as gold standard) in dynamic PET studies. This has encouraged to design and build a dedicated small PET scanner for non-invasive image-guided input function estimation (SynchroPET ArterialPETTM scanner (Stony Brook, NY, USA) [92]. Such scanners require very small FOV (as large as a human arm diameter) with a good spatial resolution to reduce errors due to partial volume effect. Low-cost, ease of use, and high spatial resolution and sensitivity for input function estimation is the incentive behind building bracelet PET scanners.

Novel PET geometries, configurations, and detector designs are proposed in the context of conceptual design which could be promising for many applications [93]. However, in some conceptual designs, manufacturing cost is ignored, and sometimes, the improvements brought by complex designs are marginal [94]. A major challenge or drawback in most dedicated PET scanners is the lack of transmission scanning and/or structural imaging. Apart from the benefits of synergistic anatomical-functional imaging to realize the full potential of quantitative PET imaging, anatomical imaging is commonly required [95]. To address this challenge, maximum-likelihood activity and attenuation (MLAA) algorithms [96], attenuation map generation using background radiation [97], and template-based attenuation map estimation approaches were designed [98]. In this regard, a major tendency consists in designing PET inserts, which could be employed on commercial MR, PET/MR, and PET/CT scanners. This could address the challenge of attenuation map generation, since anatomical/transmission data are readily available on the host scanners [99, 100].

It should be noted that owing to the astonishing performance of artificial intelligence-based algorithms, in particular deep learning methods, novel approaches for performing attenuation and scatter correction (ASC) on PET data without using anatomical images have been developed [101–103]. These include ASC in the image domain [104], attenuation correction factor estimation in the sinogram domain [104], hybrid MLAA and deep learning methods [105], and attenuation map estimation from non-ASC PET images [106]. Moreover, deep learning algorithms are employed for accurate event positioning, calibration, post-reconstruction processing, and image quality enhancement. These techniques not only boost the overall quantitative accuracy and image quality of PET scans, but could also reduce manufacturing costs [9, 107]. A recently developed maximum-likelihood attenuation correction factor (MLACF) algorithm was adapted to a dedicated brain TOF-PET scanner and implemented in the commercialized system [108]. In this method, the authors combine an MLACF method that simultaneously synthesizes the emission data and attenuation sinogram from TOF-PET data, along with a scaling technique based on anatomical features.

More aggressive efforts to achieve a coincidence time resolution of only a few tens of picoseconds and initial promising results suggest that future PET scanners can indeed rely even more on TOF to improve image quality [109]. There are ongoing debates on the technological limitations of achieving very small CTR values to reach the reconstruction-less capability. Nonetheless, more precision in TOF data leads to higher image SNR and better mitigation of limited-angle tomography.

Fortunately, in the presence of TOF, heavily compressed sinograms with axial rebinning and significant azimuthal mashing can be used without resolution loss [110]. Nevertheless, list-mode reconstruction remains a top choice for many researchers and even on commercial total-body [111, 112] and non-cylindrical PET scanners (e.g., Ref. [47]). With list-mode iterative reconstruction, an accurate physics model of the scanner, including the exact positioning of each LOR, DOI, shift-varying PSF, and TOF can be incorporated in the system matrix. Image artifacts that can be caused by asymmetrical geometries of some organ-specific dedicated PET scanners were elegantly discussed in Ref. [113], also highlighting how incorporation of a TOF can minimize such artifacts.

A new trend in high-resolution PET instrumentation includes dedicated specimen systems for intra-operative assessment of surgical samples for the assessment of lesions heterogeneity and surgical margins in three dimensions [114, 115] and organs-on-chips (OOCs) microdevices mimicking in vivo organs [116], which are finding promising applications in disease modeling and drug discovery. These developments are expected to grow in the future as there appears to be a market for these devices.

It is gratifying to see in perspective all innovative developments in PET instrumentation, from fully 3D imaging without septa to TOF, resolution recovery reconstruction, digital SiPM-based photodetectors, and more recently long axial field-of-view designs. Advances in PET instrumentation and associated image reconstruction and quantification techniques have been very swift and stimulating, and there is every reason to believe that the field will move forward more swiftly in the future with the advent of novel scintillators and photodetector technologies and the unlimited imagination of PET scientists. There is no shortage of challenges and opportunities for PET instrumentation and innovative conceptual designs.

While PET scanner technology witnessed spectacular advancements over the years, many innovative design concepts have not progressed to commercial products for various reasons. These motives can be summarized in five main aspects: fabrication cost and market readiness, service/maintenance cost, patient discomfort, suboptimal real-world performance, and difficulties associated with translating developments from academic to corporate settings. Total-body PET scanners or scanners with high temporal TOF resolution are usually expensive at the present time, which makes them less affordable in the clinic, particularly in low GDP countries [49, 107]. The extendable FOV PET design concept or compensation of the low TOF resolution through deep learning-based image quality enhancement might address this limitation [4, 8, 9]. Maintenance cost is another significant hurdle, especially for PET scanners with moveable detector configurations. Such scanners are more prone to mechanical damage, sensitive to calibration issues, and can contribute to patient discomfort, thereby increasing the maintenance cost and patient anxiety [5]. Hence, it is imperative to establish meticulous protocols for calibration and quality assurance.

Patient comfort is a fundamental consideration in PET scanner design and manufacturing. A few geometrical designs, such as spherical or dodecahedral shapes, may induce feelings of discomfort and claustrophobia [58]. Likewise, scanners with dynamic gantries could potentially cause anxiety when the detectors approach the patient [5, 117]. While these issues can be mitigated through the use of virtual reality headsets or anxiety-reducing medications, it is crucial that these factors are taken into account during the design process to ensure patient's comfort and cooperation. Another important aspect is the performance of the suggested designs in real-world scenario. Many of the suggested configurations were evaluated based on Monte Carlo simulation studies that have ignored several physical factors, which can downgrade the performance. Finally, some concepts like portable, handheld PET scanners also face significant hurdles. Despite the potential for point-of-care application, the need for radiation shielding, stringent regulatory requirements, and the difficulty of miniaturizing the necessary technology all contribute to the non-viability of these designs.

## Data Availability

The data used in this manuscript are available upon reasonable request.

## Abbreviations

PET: Positron emission tomography
AI: Artificial intelligence
DOI: Depth of interaction
TOF: Time-of-flight
LOR: Line of response
CTR: Coincidence time resolution
FWHM: Full width-at-half-maximum
LYSO: Lutetium–yttrium oxyorthosilicate
GSO: Gadolinium orthosilicate
GAGG: Gadolinium aluminum gallium garnet
LGSO: Lutetium–gadolinium oxyorthosilicate
APD: Avalanche photodiode
PSPMT: Position-sensitive photomultiplayer tube
SiPM: Silicon photomultiplier
MPPC: Multi-pixel photon counter
